# Extensive sonographic ulnar nerve enlargement above the medial epicondyle is a characteristic sign in Hansen’s neuropathy

**DOI:** 10.1371/journal.pntd.0005766

**Published:** 2017-07-28

**Authors:** Lokesh Bathala, Venkataramana N. Krishnam, Hari Kishan Kumar, Vivekananda Neladimmanahally, Umashankar Nagaraju, Himanshu M. Kumar, Johan A. Telleman, Leo H. Visser

**Affiliations:** 1 Department of Neurology, Aster CMI Hospital, Bangalore, India; 2 Department of Neurosurgery, BGS Global Hospital, Bangalore, India; 3 Department of Dermatology, Raja Rajeswari Medical College & Hospital, Bangalore, India; 4 Department of Public health, Rajiv Gandhi Institute of Public Health and Center for Disease Control, Bangalore, India; 5 Department of Neurology and Clinical Neurophysiology, Elisabeth-Tweesteden Hospital, Tilburg, The Netherlands; University of Tennessee, UNITED STATES

## Abstract

**Objective:**

Earlier studies have shown sonographic enlargement of the ulnar nerve in patients with Hansen’s neuropathy. The present study was performed to determine whether sonography or electrophysiological studies can detect the specific site of ulnar nerve pathology in leprosy.

**Methods:**

Eighteen patients (thirty arms) with Hansen’s disease and an ulnar neuropathy of whom 66% had borderline tuberculoid (BT), 27% lepromatous leprosy (LL) and 7% mid-borderline (BB) leprosy were included in the study. Cross-sectional area (CSA) of ulnar nerve was measured every two centimeters from wrist to medial epicondyle and from there to axilla. All patients underwent standard motor and sensory nerve conduction studies of the ulnar nerve. Thirty age and sex matched controls underwent similar ulnar nerve CSA measurements and conduction studies.

**Results:**

Ulnar nerve was clinically palpable in 19 of the 30 arms of patients. Motor and sensory nerve conduction studies of the ulnar nerve showed a reduced compound motor action potential and sensory nerve action potential amplitude in all patients. Motor Conduction Velocity (MCV) in patients were slower in comparison to controls, especially at the elbow and upper arm, but unable to exactly locate the site of the lesion. In comparison to controls the ulnar nerveCSA was larger in the whole arm in patients and quite specific the maximum enlargement was seen between nulnar sulcus and axilla, peaking at four centimeters above the sulcus.

**Conclusions:**

A unique sonographic pattern of nerve enlargement is noted in patients with ulnar neuropathy due to Hansen’s disease, while this was not the case for the technique used until now, the electrodiagnostic testing. The enlargement starts at ulnar sulcus and is maximum four centimeters above the medial epicondyle and starts reducing further along the tract. This characteristic finding can help especially in diagnosing pure neuritic type of Hansen’s disease, in which skin lesions are absent, and alsoto differentiate leprosy from other neuropathies in which nerve enlargement can occur.

## Author summary

Hansen’s neuropathy is caused by Mycobacterium leprae. The diagnosis of Hansen disease is based on typical skin lesions, nerve enlargement and presence of bacilli in the skin smear. One of the types of leprosy is a pure neuritic type which manifests without skin lesions. High resolution ultrasound is new modality by which the morphology of the peripheral nerves can be studied. Earlier studies have shown that peripheral nerves are thickened in Hansen’s disease and ultrasound is superior in identifying nerve enlargement when compared with clinical palpation. Good correlation exits between nerve enlargement and electrophysiological studies. The current research was to look for a specific pattern of nerve enlargement and we studied patients with Hansen’s ulnar neuropathy. We found an unique pattern of nerve enlargement in which the ulnar nerve enlargement starts at the sulcus and the enlargement is maximum four centimetres above the sulcus and then it tapers. This pattern of unique nerve enlargement can help especially in diagnosing pure neuritic type of Hansen’s disease, in which skin lesions are absent, and is also useful to differentiate leprosy from other neuropathies in which nerve enlargement can occur.

## Introduction

High-resolution ultrasonography (HRUS) is a new imaging technique to assess morphology of the peripheral nerves [1]. It has a definite role in mononeuropathies in which it complements electrophysiology [2, 3]. The most important sonographic parameter is nerve cross sectional area (CSA). Additional parameters include nerve echogenicity, vascularity and fascicular architecture. Nerve enlargement is present in several types of polyneuropathies, such as hereditary motor sensory polyneuropathy (HMSN), chronic demyelinating inflammatory polyneuropathy (CIDP), multifocal motor neuropathy (MMN), hereditary neuropathy with liability to pressure palsy (HNPP), and also in leprosy [4]. For clinicians it is important to differentiate between these diseases.

Leprosy is a chronic infectious disease caused by Mycobacterium leprae which primarily involves the skin and nerves. Nerve involvement affects sensory, motor and autonomic function of peripheral nerves [5]. Primary neuritic leprosy (PNL) is a rare variant without apparent skin lesions. It carries the highest risk of deformity, often due to late recognition [6]. Nerve involvement affects sensory, motor and autonomic function of peripheral nerves. Some nerve involvement may be seen in all types of leprosy, but leprosy reactions are the main cause of severe morbidity and can cause acute neuritis which requires immediate treatment. Nerve impairment is reversible if recognized early and treated promptly [7]. Hence, the most important goal in the management of leprosy is early detection of nerve impairment.

Currently, nerve assessment in leprosy relies mainly on clinical palpation of the nerve for nerve enlargement and on nerve conduction studies [8, 9], but these techniques, have limitations and may therefore lead to treatment delay. HRUS is an additional technique that could reduce delay in diagnosis of leprosy and of presence of leprosy reactions.In our earlier paper on Hansen’s neuropathy with electrophysiological correlations we found inverse correlation between CSA of the ulnar nerve above the elbow with Compound Motor Action Potential (CMAP), MCV across the elbow and motor weakness [10]. In other studies Color Doppler (CD) imaging of the nerves showed increased vascularization in reactions probably indicating inflammation, [11] and a study by Jain et al [12] showed that sonography is more capable to detect early nerve enlargement than clinical palpation. Chaduvula et al followed patients with Hansen’s neuropathy for two years with HRUS. They found that the nerve size and endoneural blood flow reduced in follow-up studies in patients with reactions and who were on treatment [13].

As nerve enlargement is also encountered in several other types of peripheral neuropathies the present study was designed to investigate whether there is specific pattern of ulnar nerve enlargement in neuropathy due to leprosy.

## Methodology

### Ethics statement

The study was approved by Institutional ethics committee, Rajarajeshwari Medical College and Hospital, Bangalore and all participants gave written informed consent.

### Participants

Eighteen patients (thirty arms) with Hansen’s disease classified as per Ridley and Jopling [14] classification were included. The diagnosis of leprosy was based on examination of skin patches and skin biopsy. All patients were given appropriate treatment as required based on a standard treatment protocol. For comparison thirty healthy volunteers were included. Mean age was thirty seven years and 83% were males. For normative data we recruited employees of hospital and students from the medical college. All volunteers were informed about the study and written consent was obtained. They were screened for any symptoms and signs of peripheral neuropathy. Detailed history was taken to look for any underlying history of metabolic disorders, infections and family history of neuropathy.

### Clinical examinations

All patients underwent clinical examination to assess ulnar nerve (UN) function. Patients were screened for current symptoms of lesions of the UN, i.e. numbness and paresthesia of the fourth and fifth digits of the hand, medial elbow pain, atrophy, weakness or clumsiness of the hand muscles innervated by the ulnar nerve.

Sensory testing was performed using Semmes-Weinstein monofilaments (SW). Sensory loss was considered to be present if patients were unable to perceive 2 grams of target force on the hand by SW filaments.

Motor strength of the first dorsal interosseous (FDI) and abductor digiti minimi (ADM) was tested with use of the Medical Research Council (MRC) rating scale. Muscle weakness was considered to be present when the MRC score was 4 or less.

### HRUS

HRUS of the UN was performed with Philips ultrasound machine 5–17 MHz linear array transducer by a single investigator blinded to the clinical diagnosis and results of clinical examination. Both cases and controls underwent sonographic assessment of the ulnar nerve. The ulnar nerve was traced from the wrist till the axilla with subjects in supine position and the elbow flexed at 70°. Cross-sectional area (CSA) of the nerve was determined within the inner margin of the hyper echoic rim.

CSA of the UN was obtained from the wrist and every two centimeters (cm) proximal from the wrist over a distance of eight cm; U1 to U4 representing every two cm from the wrist. Measurement was then performed at ulnar sulcus and from there every two cm proximal to sulcus; U5 and U6 and at axilla (U7).

CSA of upper, middle and lower trunks of the brachial plexus was measured on the side of the examined limb for both cases and controls. Normative data of CSA of ulnar nerve has been published in our earlier paper [15].

To determine vascularization Color Doppler (CD) setting were chosen to optimize identification of weak signals from vessels with slow velocity. The PRF (Pulse Repetition Frequency) used was 1 kHz and the band filter was set at 50 Hz. The presence of blood flow signals in epineural plexus or endoneural vessels would indicate hypervascularity of the nerve during CD imaging.

#### Electrophysiology

All cases and controls underwent standardized nerve conduction studies of ulnar nerve as per the guidelines of the American Association of Neuromuscular &Electro diagnostic Medicine (AANEM).Details of the nerve conduction studies protocol have been described in our earlier paper on normative data for CSA of ulnar nerves [15].

### Statistical analysis

Statistical analysis was conducted with use of SPSS20.0, IBM Corporation. NerveCSA, CMAP, MCV, SNAP, SNCL and DSL were treated as outcome variables while demographic and clinical data were treated as co-variables. All outcome variables were log-transformed to approximate normal distribution as variables were skewed and to be used for inferential statistics. Co-relation of continuous co-variables with outcome variables was tested using Pearson correlation test while relation of categorical co-variables with outcome variables was tested using multivariate general linear models. Difference in means of nerve CSA and electrophysiological variables between cases and controls was tested using independent sample‘t’ test. Bonferroni’s correction was applied as difference in means was tested on repeated measures on the same limb. Association between electrophysiology measures and cross sectional area of nerve in patients and controls was tested using linear regression analysis. Difference between lower 95% confidence limit (95% CL) of higher means and higher 95% CL of lower means was estimated for all significantly different means to identify the largest point(s) of difference between two groups. Means of cases and controls with 95% confidence interval (95% CI) for nerve CSA and nerve conduction measurements were plotted graphically to identify patterns of variation. Statistical results with p value less than 0.05 were considered significant.

## Results

Eighteen patients (thirty arms) with Hansen’s disease with mean age 37.6± 12.3 years, range 24 to 70 years were included in the study. Eighty six percent of patients were male and 66% were classified as having borderline tuberculoid (BT), 27% lepromatous leprosy (LL), and 7% mid-borderline (BB) leprosy. On clinical examination motor weakness of ADM and FDI (MRC grade less than 4) was found in 93.3% and reduced sensation in ulnar innervated area in 90%.Ulnar nerve was thickened on palpation in 63.3% of cases. In the thirty age and sex matched controls mean age was 36.97 (±12) years and 83% was male.

### Neurophysiology

**Fig 1:**
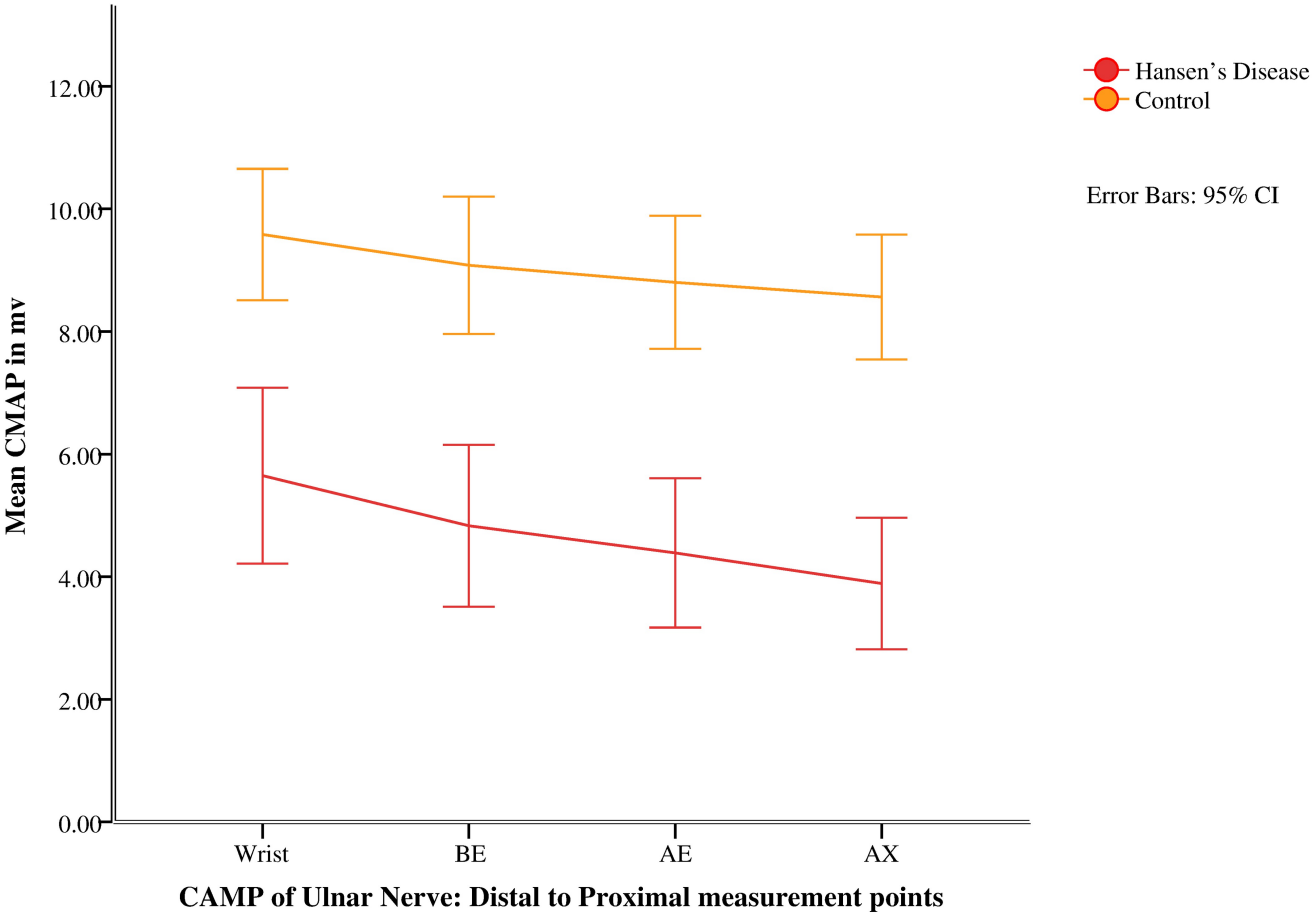
Distal to proximal mean CAMP of ulnar nerve in patients with Hansen’s disease and healthy controls.

**Fig 2:**
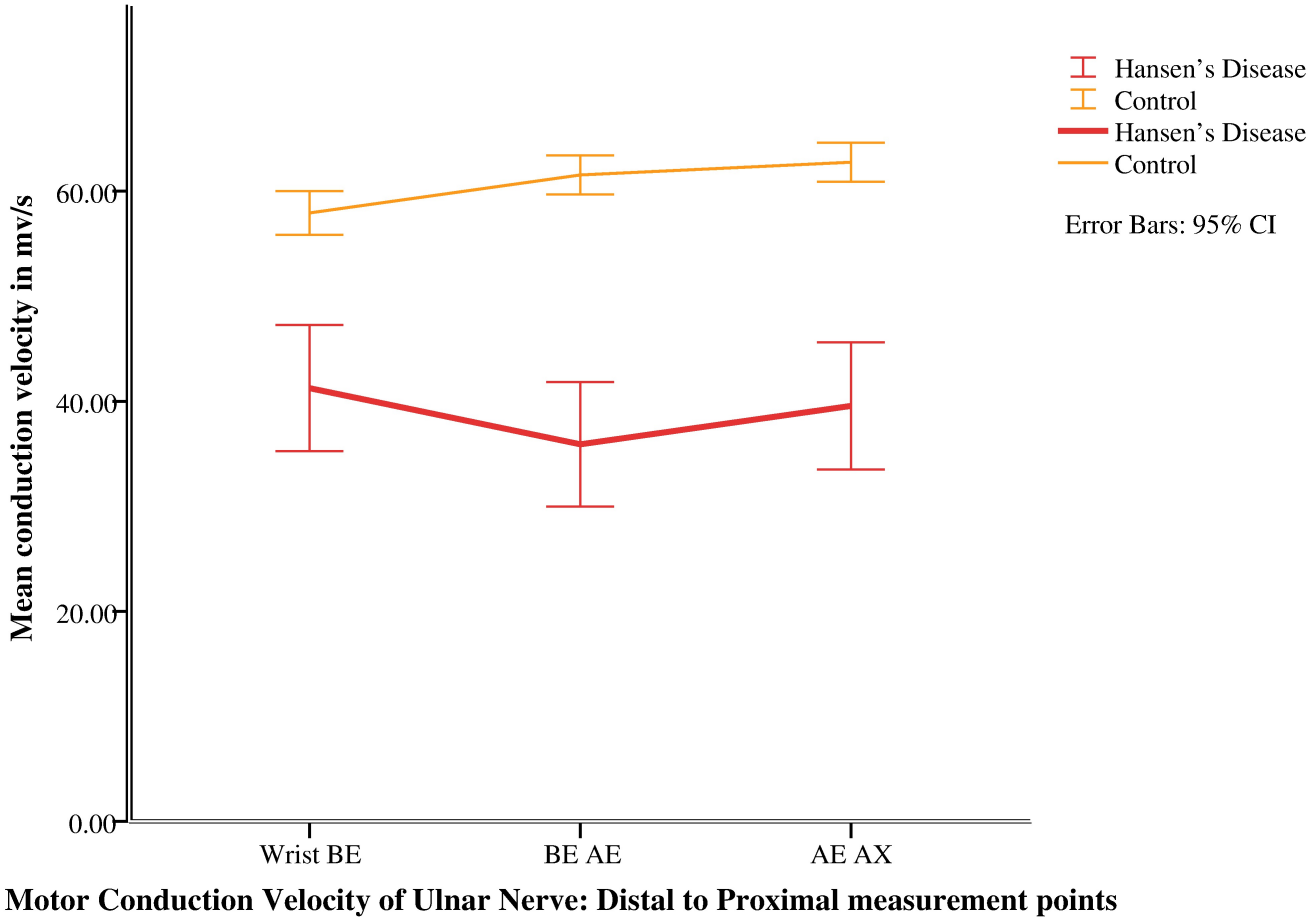
Distal to proximal mean conduction velocity of ulnar nerve in patients with Hansen’s disease and healthy controls.

Two patients did not consent for nerve conduction studies, hence conduction measurements of 26 upper limbs were analyzed. Neurophysiological values of cases and controls are summarized in Table 1. Mean CMAP (Compound Motor Action Potential) and MCV (Motor Conduction Velocity) of cases were significantly lower than that of controls at all points of measurements. Mean CMAP and mean MCV of cases were lowest at AE (Above elbow) and BE-AE (below elbow- above elbow) respectively (Figs 1 & 2).Likewise, mean SNAP (sensory nerve action potential) and mean SNCV (sensory nerve conduction velocity) were significantly lower than that of controls (p<0.05). The mean distal motor latency was significantly longer in cases than controls (p<0.05, Table 1).

**Table 1:** Electrophysiological values comparing healthy controls and patients with Hansen’s neuropathy.

	Cases	Controls
Distal motor latency m/sec	3.64^#^ (1.53) *	2.40 (0.39)
CMAP (mv)	5.65^#^ (3.55) *	9.58 (2.87)
CMAP BE (mv)	4.83^#^ (3.26) *	9.08 (3.00)
CMAP AE (mv)	4.39 ^#^ (3.02) *	8.80 (2.91)
CMAP AX (mv)	3.89^#^ (2.66) *	8.56 (2.73)
MCV wrist m/sec	41.26^#^ (14.89) *	57.93 (5.55)
MCV BE AE m/sec	35.92^#^ (14.71) *	61.57 (4.99)
MCVAE AX m/sec	39.56^#^ (14.99) *	62.77 (5.02)
Distal Sensory Latency m/sec	2.00 (1.13) *	2.11 (0.27)
SNAP m/sec	5.25^#^ (5.27) *	16.70 (8.70)
SNCV m/sec	35.60^#^ (19.15) *	54.03 (5.01)

CMAP, Compound motor action potential; MCV, Motor conduction velocity; SNAP, Sensory nerve action potential; SNCV, Sensory nerve conduction velocity; BE-AE, below elbow- above elbow; AE-AX, above elbow- axilla;

^#^—Significantly different from mean of controls with p<0.05 as measured by independent sample’t’ test, p values are adjusted for Bonferroni’s correction;

*—n = 26.

### Sonography

**Fig 3:**
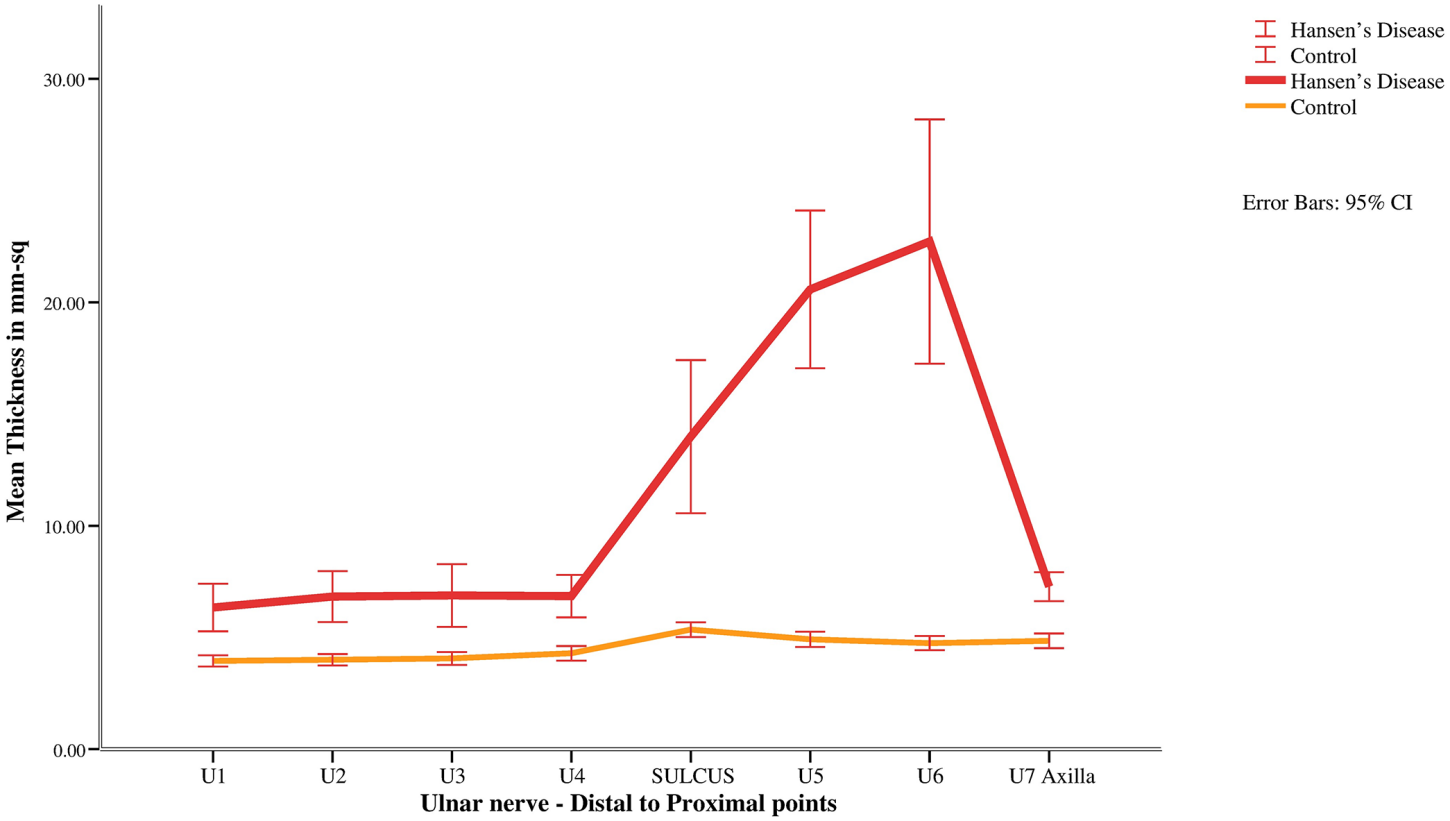
Distal to proximal mean thickness of ulnar nerve (CSA- mm^2^) in patients with Hansen’s disease and healthy controls.

All patients underwent nerve ultrasound. Mean CSA of ulnar nerve was significantly higher than that of controls at all points of measurements with a hump between sulcus and axilla, peaking at point U6, which is four centimeters from the sulcus (Fig 3). Linear regression analysis showed negative association between CSA at U6 and CMAP, MCV and latency measures cases. (Table 2). Among cases 67% showed increased vascularity on color Doppler, 53% had moderate and 47% had severe disruption of fascicular architecture.

**Table 2:** Regression analysis of CSA at U6 with electrophysiological values.

Sl no.	Predictor	Standardized β	p value
	**Model 1—R**^**2**^ = **0.35 p = 0.01**		
1.	CMAP above elbow	-0.469	0.026
2.	MCV above elbow	-0.211	NS
	**Model 2—R**^**2**^ = **0.39 p = 0.01**		
1.	Distal Motor Latency	-0.406	0.04
2.	Distal Sensory Latency	-0.426	0.03
	Model 3- NS		
1.	SNAP	-0.347	NS
2.	SNCV	0.113	NS
	**Model 4—R**^**2**^ = **0.69 p = 0.008**		
1.	CMAP above elbow	-0.201	NS
2.	MCV above elbow	-0.453	0.03
3.	Distal Motor Latency	-0.527	0.08
4.	Distal Sensory Latency	-0.725	0.05
5.	SNAP	0.193	NS
6.	SNCV	-0.285	NS

Standardized β estimates are derived from linear regression analysis. CMAP—Compound Motor Action Potential, MCV—Conduction Velocity, SNAP—sensory nerve action potential, SNCV—sensory nerve conduction velocity, NS- not significant

## Discussion

**Fig 4:**
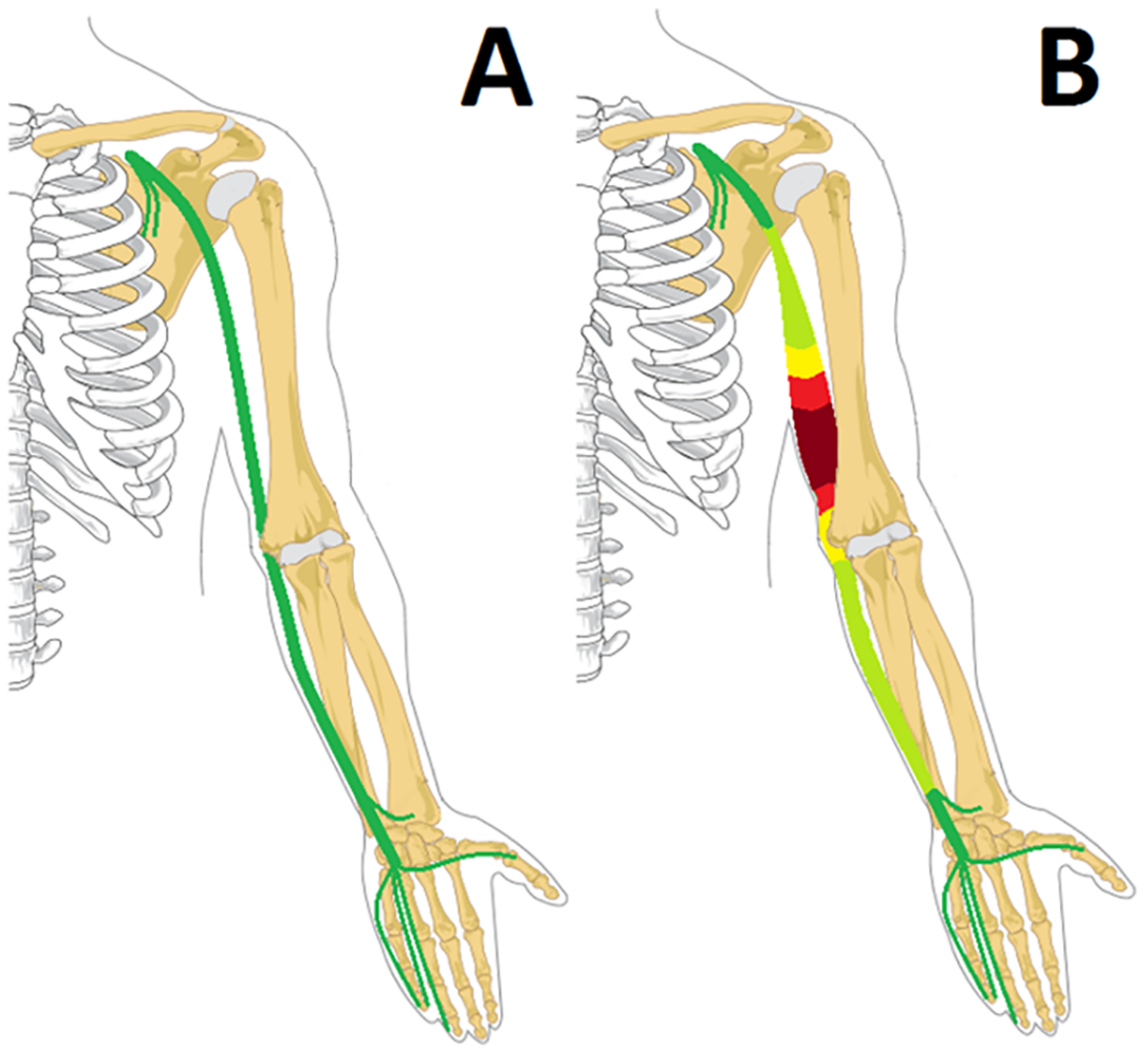
Pattern of nerve enlargement in leprosy. A—Normal ulnar nerve. B—Maximum enlargement few centimetres proximal to sulcus.

We undertook the present study to look for a specific pattern of ulnar nerve pathology in skin biopsy proven cases of Hansen’s by using the standard technique of nerve conduction studies and secondly the imaging of the ulnar nerve by sonography. Motor and sensory nerve conduction studies showed a reduced CMAP and SNAP amplitude in all patients. Motor conduction velocities were slower in comparison to controls, especially at the elbow and upper arm, but with electro diagnostic testing we were unable to exactly locate the site of the lesion. With ultrasound the result was striking as in all of the patients the nerve was enlarged at all levels compared to controls. However, the most significant increase in CSA was observed a few centimeters above the sulcus, while CSA came to near normal values near the axilla (Figs 3 and 4).

Nerve enlargement is not only found in leprosy, but also in other types of (demyelinating) neuropathy. In CMT, and especially CMT1A, diffuse, massive enlargement of all nerves is found [16, 17, 18], while in HNPP nerve enlargement is mainly found at entrapment sites [19]. In CIDP, nerve enlargement is often to a lesser degree than in CMT, and most often proximal nerve segments are involved [20]. Though nerve enlargement is present in all those conditions, our study showed a unique pattern of enlargement in leprosy, with maximum enlargement proximal to ulnar sulcus. This sonographic feature may therefore be very useful to discriminate leprosy from those other types of neuropathy.

One of the challenges in leprosy is pure neuritic type of Hansen’s which constitutes 4–18% of leprosy cases. Its uniqueness is absence of skin lesions and therefore diagnosing this condition can be a challenge. Jain et al [21] described a patient of pure neuritic Hansen’s affecting the ulnar nerve. Sonography showed similar nerve enlargement as patients with skin lesions, and the diagnosis was confirmed by dorsal ulnar cutaneous nerve biopsy. As PNL shows sonographic nerve enlargement, and our study showed a unique pattern of enlargement, performing sonographic examination in addition to EDX could help the clinicians in diagnosing PNL in patients presenting with ulnar neuropathy without any skin lesions, and could therefore help avoid nerve biopsies in those patients.

### Conclusions

A unique pattern of nerve enlargement is noted in all patients with ulnar neuropathy due to Hansen’s disease. The enlargement starts at the sulcus and is maximum four centimeters above the medial epicondyle after which it starts reducing. Identifying this unique pattern of nerve enlargement could help in diagnosing pure neuritic type of Hansen’s disease where skin lesions are absent and could also help to discriminate Hansen’s disease from other neuropathies associated with sonographic nerve enlargement.

## References

[1] Beekman R, Visser LH. (2004). High-resolution sonography of the peripheral nervous system: a review of the literature. Eur J Neurol; 11:305–314. doi: 10.1111/j.1468-1331.2004.00773.x 15142223 10.1111/j.1468-1331.2004.00773.x

[2] Padua L, Aprile I, Pazzaglia C, et al Contribution of ultrasound in a neurophysiological lab in diagnosing nerve impairment: a one-year systematic assessment. Clin Neurophysiol 2007; 118:1410–1416. doi: 10.1016/j.clinph.2007.03.011 17466584 10.1016/j.clinph.2007.03.011

[3] Padua L, Hobson-Webb LD, Martinoli C. Nerve conduction and ultrasound: will the wedding give birth to new morpho-functional measures. Clin Neurophy siol 2010; 121:130–131. 10.1016/j.clinph.2009.10.014 19955016

[4] Khadilkar SV, Yadav RS, Soni G. A practical approach to enlargement of nerves, plexuses and roots. Pract Neurol. 2015 4; 15(2):105–15. doi: 10.1136/practneurol-2014-001004 25573343 10.1136/practneurol-2014-001004

[5] Britton WJ, Lockwood DN. Leprosy. Lancet 2004; 363:1209–1219. doi: 10.1016/S0140-6736(04)15952-7 15081655 10.1016/S0140-6736(04)15952-7

[6] Rao PN, Suneetha S. Pure neuritic leprosy: Current status and relevance. Indian J Dermatol Venereol Leprol. 2016 May-Jun; 82(3):252–61. doi: 10.4103/0378-6323.179086 27088926 10.4103/0378-6323.179086

[7] Job CK. Nerve damage in leprosy. Int J Lepr Other Mycobact Dis 1989; 57:532–539. 2545801

[8] Van Brakel WH, Nicholls PG, Das L, Barkataki P, Maddali P, Lockwood DNJ et al (2005). The INFIR conhort study: assessment of sensory and motor neuropathy in leprosy at baseline. Lepr Rev; 76:277–295. 16411508

[9] Van Brakel WH, Nicholls PG, Wilder-Smith EP, et al; on behalf of the INFIR Study Group. Early diagnosis of neuropathy in leprosy comparing diagnostic tests in a large prospective study (the INFIR Cohort Study). PLoS Negl Trop Dis 2008; 2:e212 doi: 10.1371/journal.pntd.0000212 18382604 10.1371/journal.pntd.0000212 PMC2270341

[10] Bathala L, Kumar K, Pathapati R, Jain S, Visser LH. Ulnar neuropathy in Hansen’s disease: clinical, high-resolution ultrasound and electrophysiologic correlations. J Clin Neurophysiol. 2012 4; 29(2):190–3. doi: 10.1097/WNP.0b013e31824d969c 22469686 10.1097/WNP.0b013e31824d969c

[11] Martinoli C, Derchi LE, Bertolotto M, Gandolfo N, Bianchi S, Fiallo P, Nunzi E (2000). US and MR imaging of peripheral nerves in leprosy. Skeletol Radiol; 29:142–150. 10.1007/s002560050584 10794551

[12] Jain S, Visser LH, Praveen TLN, et al High resolution sonography: a new technique to detect nerve damage in leprosy. PLoS Negl Trop Dis. 2009; 8:e498. 10.1371/journal.pntd.0000498 PMC2716078 19668356

[13] Chaduvula MV, Visser LH, Suneetha S, Suneetha L, Devaraju B, Ellanti R, Raju R, Jain S. High-Resolution Sonography as an Additional Diagnostic and Prognostic Tool to Monitor Disease Activity in Leprosy: A Two-Year Prospective Study. Ultraschall Med. 2016; 6 7. 10.1055/s-0042-108430 27273176

[14] Ridley D S, Jopling W H (1966). Classification of leprosy according to immunity: a five-group system. Int. J. Leprosy 34:255–73. 5950347

[15] Bathala L, Kumar P, Kumar K, Visser LH. Ultrasonographic cross-sectional area normal values of the ulnar nerve along its course in the arm with electrophysiological correlations in 100 Asian subjects. Muscle Nerve. 2013 5; 47(5):673–6. doi: 10.1002/mus.23639 23401025 10.1002/mus.23639

[16] Zaidman CM, Harms MB, Pestronk A. Ultrasound of inherited vs. acquired demyelinating polyneuropathies. J Neurol. 2013 12; 260(12):3115–21. doi: 10.1007/s00415-013-7123-8 24101129 10.1007/s00415-013-7123-8 PMC3970398

[17] Goedee HS, Brekelmans GJ, Visser LH. Multifocal enlargement and increased vascularization of peripheral nerves detected by sonography in CIDP: a pilot study. Clin Neurophysiol. 2014 1; 125(1):154–9. doi: 10.1016/j.clinph.2013.05.025 23880223 10.1016/j.clinph.2013.05.025

[18] Sugimoto T, Ochi K, Hosomi N, Takahashi T, Ueno H, Nakamura T, Nagano Y, Maruyama H, Kohriyama T, Matsumoto M. Ultrasonographic nerve enlargement of the median and ulnar nerves and the cervical nerve roots in patients with demyelinating Charcot-Marie-Tooth disease: distinction from patients with chronic inflammatory demyelinating polyneuropathy.J Neurol. 2013 10;260(10):2580–7. doi: 10.1007/s00415-013-7021-0 23821028 10.1007/s00415-013-7021-0

[19] Goedee SH, Brekelmans GJ, van den Berg LH, Visser LH. Distinctive patterns of sonographic nerve enlargement in Charcot-Marie-Tooth type 1A and hereditary neuropathy with pressure palsies. Clin Neurophysiol. 2015 7; 126(7):1413–20. doi: 10.1016/j.clinph.2014.08.026 25454274 10.1016/j.clinph.2014.08.026

[20] Jang JH, Cho CS, Yang KS, Seok HY, Kim BJ. Pattern analysis of nerve enlargement using ultrasonography in chronic inflammatory demyelinating polyneuropathy. Clin Neurophysiol. 2014 9; 125(9):1893–9. doi: 10.1016/j.clinph.2013.12.115 24560630 10.1016/j.clinph.2013.12.115

[21] Jain S, Visser LH, Yerasu MR, Raju R, Meena AK, Lokesh B, Suneetha S. Use of high resolution ultrasonography as an additional tool in the diagnosis of primary neuritic leprosy: a case report. Lepr Rev. 2013 Jun; 84(2): 161–5. 24171244

